# Molecular Pathophysiology of Autosomal Recessive Polycystic Kidney Disease

**DOI:** 10.3390/ijms22126523

**Published:** 2021-06-17

**Authors:** Adrian Cordido, Marta Vizoso-Gonzalez, Miguel A. Garcia-Gonzalez

**Affiliations:** 1 Grupo de Xenética e Bioloxía do Desenvolvemento das Enfermidades Renais, Laboratorio de Nefroloxía (No. 11), Instituto de Investigación Sanitaria de Santiago (IDIS), Complexo Hospitalario de Santiago de Compostela (CHUS), 15706 Santiago de Compostela, Spain; adriancosman@hotmail.es (A.C.); martavizosoglez@gmail.com (M.V.-G.); 2 Grupo de Medicina Xenómica, Complexo Hospitalario de Santiago de Compostela (CHUS), 15706 Santiago de Compostela, Spain; 3 Fundación Publica Galega de Medicina Xenómica-SERGAS, Complexo Hospitalario de Santiago de Compostela (CHUS), 15706 Santiago de Compostela, Spain

**Keywords:** ARPKD, cyst, rare monogenic disease, nephrology

## Abstract

Autosomal recessive polycystic kidney disease (ARPKD) is a rare disorder and one of the most severe forms of polycystic kidney disease, leading to end-stage renal disease (ESRD) in childhood. *PKHD1* is the gene that is responsible for the vast majority of ARPKD. However, some cases have been related to a new gene that was recently identified (*DZIP1L* gene), as well as several ciliary genes that can mimic a ARPKD-like phenotypic spectrum. In addition, a number of molecular pathways involved in the ARPKD pathogenesis and progression were elucidated using cellular and animal models. However, the function of the ARPKD proteins and the molecular mechanism of the disease currently remain incompletely understood. Here, we review the clinics, treatment, genetics, and molecular basis of ARPKD, highlighting the most recent findings in the field.

## 1. Introduction

Autosomal recessive polycystic kidney disease (ARPKD) is a severe inherited cystic disease characterized by the combination of bilateral renal cystic disease and congenital hepatic fibrosis. ARPKD manifests perinatally, or in childhood, as an important cause of pediatric morbidity and mortality [1]. ARPKD is a rare disease; an incidence of 1 in 8000 births was calculated in an isolated and inbred population from Finland [2]. However, in the Americas (North, Central, and South), the reported incidence is 1 in 26:485 births, and the annualized prevalence is 1.17 per 100,000 [3]. The widespread prevalence of ARPKD is estimated to be 1 in 20:000 births [4].

ARPKD is the recessive form of a group of heterogeneous monogenic disorders named polycystic kidney disease (PKD). The dominant form, autosomal dominant polycystic kidney disease (ADPKD), has a higher epidemiological prevalence and is typically diagnosed in adults [5].

## 2. Autosomal Recessive Polycystic Kidney Disease Clinical Presentation

ARPKD is phenotypically highly variable; it can present as a disease of perinatal, neonatal, infantile, juvenile, or young adult-onset disease [6], with no known gender or ethnic bias [7]. Typically, the most severe cases of ARPKD present in the late gestational or neonatal stage, with bilateral massively enlarged and echogenic kidneys with poor corticomedullary differentiation, retained reniform contour, and multiple tiny cysts [8,9,10]. In addition, they can present with oligo- or anhydramnios, resulting in the typical “Potter sequence” phenotype with pulmonary hypoplasia, characteristic facial features, and clubfoot contracted limbs [9,11,12]. In addition to the Potter sequence, the presence of other extrarenal manifestations is not common [13]. There are no documented hepatic phenotypes [14], although some associated, such as abdominal dystocia, have been reported [15].

Detection of severe oligohydramnios is associated with worse outcome due to the high risk of associated pulmonary hypoplasia. Up to 50% of ARPKD neonates die of respiratory insufficiency due to pulmonary hypoplasia and thoracic compression. However, after the perinatal period, survival is high, reaching 1-year and 10-year survival rates of 85% and 82%, respectively [7,8,12]. The patients who survive the perinatal period require extensive care by specialists in internal medicine [16]. Note that prenatal diagnosis and termination of pregnancy are factors to consider in the epidemiology of the disease [15,17]. Another problem derived from kidney enlargement and pulmonary hypoplasia, in addition to early uremia and pulmonary immaturity, includes the difficulty of enteral feeding that could complicate nutrition, requiring persistent nasogastric feeding [11,18].

In most cases, the severe problem of ARPKD during the first few months of life is arterial hypertension, which requires treatment with multiple classes of pharmacological interventions. Tight blood pressure control is crucial to prevent further kidney damage from hypertension [11,12]. However, renal failure is not a very common cause of neonatal demise [19]. Renal disease manifestations include urinary tract infections, macroscopic hematuria, as well as renal osteopathy early in childhood. In regards to sonography anomalies, increased echogenicity and renal cysts are usually reported prior to kidney enlargement [14]. Kidney enlargement is due to extensive dilatations of the distal nephron, typically in the collecting duct. As the disease progresses, the kidney structure gradually resembles the pattern seen in ADPKD with renal macrocysts of different size and appearance, often accompanied by interstitial fibrosis [11,20,21]. This situation leads to almost 50% of end-stage renal disease (ESRD) in the first decades of life, requiring renal replacement therapy [9,14,17].

Although the disease is called “autosomal recessive polycystic kidney disease,” a cystic liver phenotype plays a significant role in the disease, which explains why the primary gene is entitled “polycystic kidney and hepatic disease 1 (*PKHD1*)”, with polycystic liver disease (PLD) being the principal extrarenal manifestation [22]. These histological changes are the consequences of a developmental defect of the hepatic ductal plate termed, ductal plate malformation (DPM) [23,24]. DPM is also a common feature of other ciliopathies [4]. Liver disease appears with increasing age of the patient. The first manifestation appears as congenital hepatic fibrosis (CHF) with variable dilatations of both intra- and extrahepatic bile ducts (Caroli syndrome) [23]. Over the course of ARPKD, liver disease presents with two main manifestations: portal hypertension due to progressive hepatic fibrosis; and cholangitis [14]. Complications related to portal hypertension can include splenomegaly, thrombocytopenia, and esophageal varices, producing severe bleeding complications [14,25]. There is some link between adult patients with ARPKD (more than 40 years) and the risk of developing hepatic tumors, especially cholangiocarcinoma [24]. Interestingly, the hepatocellular function normally remains stable, with serum liver enzymes in the normal range, except for cholestasis parameters [4,12,23,26]. With the current clinical methods, this fact makes it difficult to monitor the severity and progression of liver disease [25].

Although cerebral aneurysms can occur in ~10% of ADPKD patients, only a few cases have been described in ARPKD [27]. The highest risk factor for an intracranial aneurysm is hypertension, a condition shared in patients with ADPKD and ARPKD [28]. The early age at which some of these aneurysms are diagnosed is remarkable since pediatric aneurysms are very rare [27]. Moreover, other extrarenal and extrahepatic manifestations can be present in ARPKD patients, including left ventricular hypertrophy, recurrent respiratory infections, neurological abnormalities, abnormal ocular fundus, abdominal pain, septic episodes, and deformities of the spine and limbs [14,29].

While a majority of ARPKD patients show similar disease progression, there are atypical phenotypes; among the elderly population, some ARPKD cases were reported as moderately affected or even exclusive or predominant phenotypes of either the liver or kidneys [4,21]. This is related to the fact that although ARPKD is a recessive disease, in which heterozygous carriers should not show any clinical manifestation of the disease, the data suggest heterozygous for *PKHD1* mutations have an increased risk of PLD and mild PKD [30,31].

## 3. Diagnosis

ARPKD is frequently diagnosed in the prenatal period due to its early and severe manifestations. In prenatal diagnosis, an ultrasound from second/third trimester can detect enlarged, echogenic kidneys, and medullary hyperechogenicity, due to the loss of corticomedullary differentiation and diffusively increased hepatic parenchymal echogenicity with fibrous tissue. The presence of oligohydramnios can make it challenging, so ultrasonography and MRI are required. The finding of microcysts (5–7 mm) was reported in 30% of ARPKD cases, but macrocysts (>10 mm) are rare and could indicate another different ciliopathy. These ultrasound findings are common in other pathologies, like Meckel syndrome, and mild forms of the disease may not be detected by prenatal ultrasounds. In these cases, the genetic test offers the possibility of providing an accurate diagnosis [32,33].

Identifying the *PKHD1* gene made it possible to perform the genetic diagnosis by direct DNA sequencing (Sanger method). However, the genetic test for the *PKHD1* mutation is complicated due to the large genomic size and the allelic heterogeneity of the disease-associated mutations [34]. However, according to the Genetics Work Group, single-gene testing should be avoided in cases of suspected ARPKD due to its broad overlapping phenotypic spectrum. As an alternative, methods such as next generation sequencing have become of interest as techniques that can simultaneously and efficiently analyze multiple candidate genes in a unique test, at relative low cost. In rare cases, mutations in two genes can even be observed in children with severe neonatal clinical phenotype [4,33,35].

The outcome of the genetic testing is essential for clinical management of comorbidities and complications associated with each disease, allowing informed genetic counselling and, in the future, precision medicine on a more specific basis [4].

## 4. Differential Diagnosis

**Figure 1:**
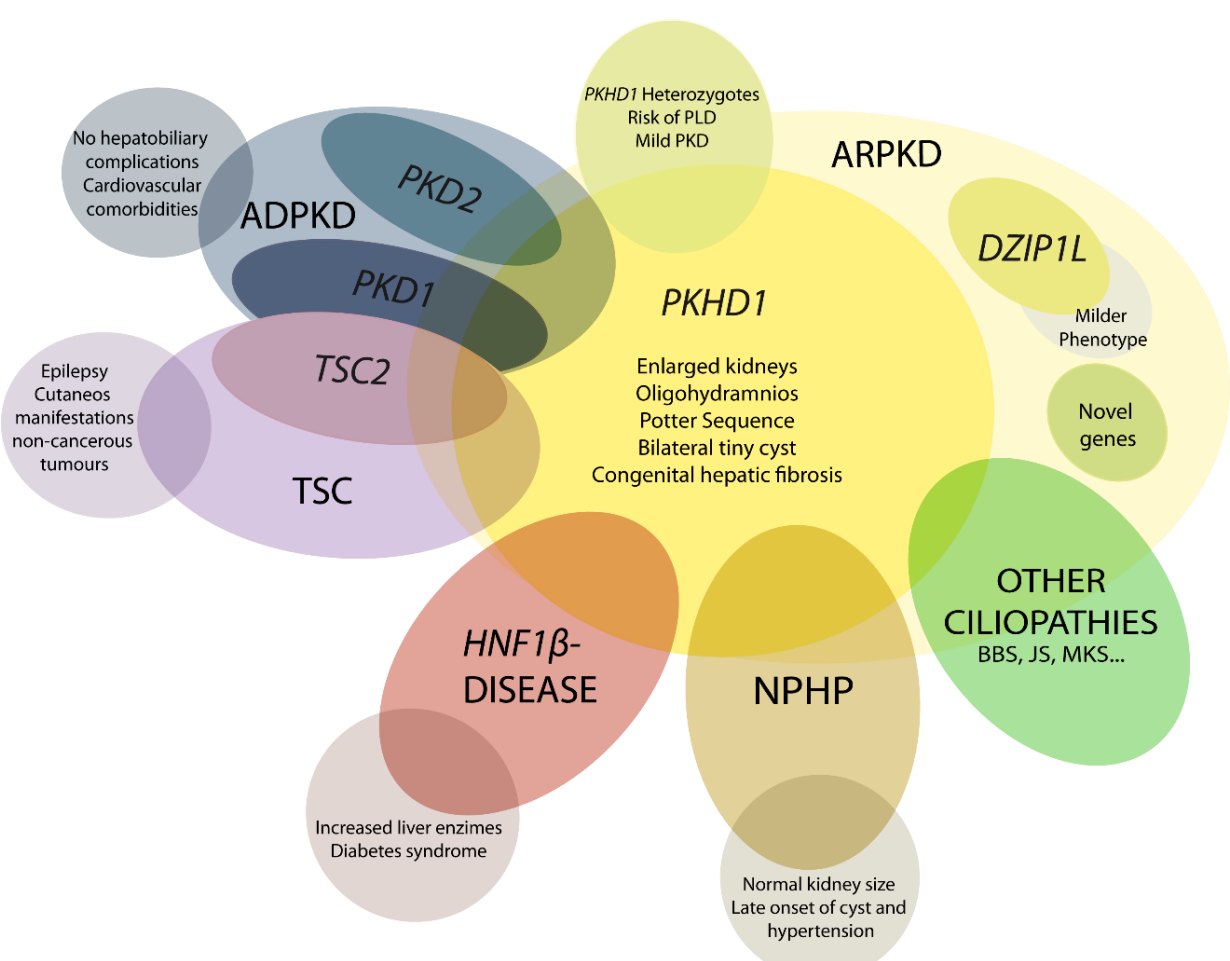
Schematic representation of ARPKD differential diagnosis. *PHKD1* is the main causative gene in ARPKD, where *DZIP1L* present milder phenotype. Mutations in other genes can overlap clinical manifestations of ARPKD, such as *PKD1* and *PKD2*, the main causative genes of autosomal dominant polycystic kidney disease (ADPKD); TSC2, that causes tuberous sclerosis (TSC); and others for instance *HNF1β*, nephronophthisis (NPHP) genes and other ciliopathies as Bardet–Biedl (BBS), Joubert (JS), and Meckel syndrome (MKS). These overlapping phenotypes manifest the physiologic complex and functional interactions that occur among ciliopathy genes.

The ARPKD phenotype is not only caused by mutations in *PKHD1*. This makes diagnosis and management, including care during the perinatal period, a difficult task. A number of other recessive and dominant genes need to be considered (Figure 1) [4,21,36].

### 4.1. DZIP1L-Related Polycystic Kidney Disease

A moderate manifestation of ARPKD has been described in patients with *DZIP1L* mutations [37]. The first reported cases associated with this gene are in the prenatal stage or in early childhood; however, no cases of perinatal death have been described [4,21,37].

### 4.2. Early and Severe Autosomal Dominant Polycystic Kidney Disease (ADPKD)

In most cases, ADPKD does not appear clinically before adulthood, but there is a small portion of cases (up to 5%) where the disease can manifest during childhood or before birth. There is no established reason that stochastic, epigenetic, and environmental aspects are believed to alter the phenotype. Families with one child showing early-onset ADPKD usually have high repetition in other siblings, leading us to believe a familial modifier, such as complex genetic interactions with second modifiers. A “dosage-sensitive network” could worsen cell integrity and could explain the more severe clinical course of these patients [4,21,38,39].

Clinically, there is a significant overlap between ARPKD and ADPKD, but there are also several characteristic manifestations associated with each disease. ADPKD patients rarely develop hepatobiliary complications, unlike patients with ARPKD. In addition, ADPKD patients are more likely to have cardiovascular comorbidities, particularly intracranial aneurysms [21].

### 4.3. Early and Severe PKD due to TSC2-PKD1

Mutations in *TSC1* and *TSC2* genes cause tuberous sclerosis (TSC). This multiorgan disorder is predominantly associated with epilepsy and cutaneous manifestations. Patients may develop non-cancerous tumors in the kidney, heart, and brain. Renal manifestations are the main cause of death in adult patients [40,41]. When a deletion in chromosome 16p affects the two adjacent genes (*PKD1* and *TSC2*), it usually causes an early onset of PKD. Moreover, their close physiologic interrelations can explain the clinical overlap between PKD and TSC; *TSC2* protein function has been shown to play a role in assisting polycystin 1 localization [21].

### 4.4. HNF1β-Related Disease

Mutations in the *HNF1β/TCF2* gene cause prenatal hyperechogenic kidneys and Potter’s sequence, as well as enlarged polycystic kidneys that can be confused with ARPKD. One explanation linking these similar phenotypes is that *HNF1β* is a transcription factor that regulates *PKHD1* expression, in addition to other polycystic kidney genes. However, mutations in this gene can cause a wide range of manifestations: renal cysts and diabetes syndrome; defects in the genital tract; endocrine/exocrine gland disorders, hypomagnesemia, and an increase in liver enzymes [21,42,43].

### 4.5. Nephronophthisis (NPHP)

NPHP is classified as an autosomal recessive cystic kidney disease that is characterized by tubulointerstitial cysts accompanied with fibrosis. To date, about 20 genes are related with recessive NPHP [36]. Unlike ARPKD, NPHP kidneys remain small. It is common that cysts and hypertension only manifest in late disease and it is one of the main causes of ESRD in patients under 25. In some cases, the manifestations may mimic ARPKD with enlarged kidneys or Potter’s sequence. NPHP proteins work in functional networks with other ciliopathy proteins; the identification and characterization of NPHP genes has contributed to the understanding of the molecular mechanisms of cystogenesis [4,21,44,45].

Medullary cystic kidney disease (recently named tubulointerstitial kidney disease TKD), caused by mutations in *MUC1* and *UMOD*, is considered the autosomal dominant NPHP, with a later onset than the recessive form [21].

### 4.6. Mutations in Other Ciliary Genes

Mutations may mimic ARPKD in genes that typically cause other (usually more complex) ciliopathies, such as Bardet–Biedl (BBS), Joubert (JS), and Meckel syndrome (MKS). BBS phenotype can be heterogeneous, but often presents enlarged and hyperechogenic kidneys with a loss of corticomedullary differentiation. The most severe ciliopathies are MKS and JS, characterized by early-onset developmental disorder and neurological problems, and many features of ciliopathy, such as liver fibrosis, polydactyly, and cystic kidneys [4,21].

Another example is Von Hippel–Lindau, an autosomal dominant disease caused by mutations in the VHL gene, a tumor suppressor gene. This causes hemangioblastoma in the central nervous system accompanied by renal tumors. Affected people also have a high probability of renal and pancreatic cysts. The similarity between the manifestations between TSC, VHL, and PKD suggests a functional connection: primary cilium and mTOR signaling pathway [4,46].

## 5. Genetics of ARPKD

**Figure 2:**
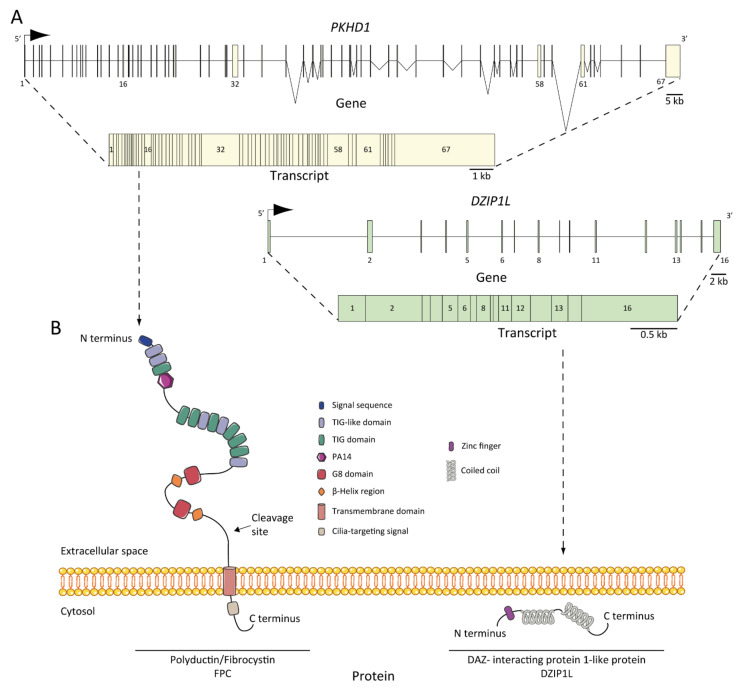
ARPKD genes, transcripts, and proteins: (**A**) *PKHD1* and *DZIP1L* genes and transcripts. The positions of the exons are illustrated and numbered, and the longest transcript are shown from both: 67 exons for *PKHD1* and 16 for *DZIP1L*; (**B**) structure of fibrocystin/polyductin (FPC) and DAZ-interacting protein 1-like protein (DZIP1L). Proteins are not to scale.

As we mentioned earlier, ARPKD is caused by mutations in *PKHD1* and, the recently discovered, *DZIP1L* [37,47,48]. *PKHD1*, located on chromosome 6 (6p12.3-p12.2) (Figure 2A) [34,48,49,50], is one of the largest human genes with a genomic segment of ~500 kb. It is predicted to have a minimum of 86 exons assembled in a complicated pattern of alternative splice variants, transcribing a large full-length mRNA of approximately 8.5 kb–13 kb [49]. Multiple types of mutations characterized as pathogenic have been identified across the gene. Currently, approximately 750 *PKHD1* mutations have been identified, of which approximately half are missense changes. A missense mutation in exon 3 (c. 107C>T; p.Thr36Me) is the most common mutation described, accounting for more than 20% of all cases [34,51]. This mutation has been observed in the context of heterozygotes, with a second distinct mutant allele [29]. Most cases are familial, but de novo mutations are also reported and account for 2 to 5% of cases [34]. Interestingly, in the context of isolated autosomal dominant polycystic liver disease (ADPLD), Besse and colleagues have reported several individuals with *PKHD1* mutations in heterozygote carriers, 10 of 102 ADPLD patients of their cohort were explained by *PKHD1* mutations, one of them presented the p.Thr36Me missense variant [30]. According to the clinical observation, it is a genetic fact that 10% of ARPKD patients present innumerable asymptomatic liver cysts [31]. However, the data are not sufficient to explain why *PKHD1* in ARPKD leads to severe hepatic and renal phenotype, but not in ADPLD; in this regard, more studies are needed [52].

Recently, using genome-wide analysis of SNPs, whole-exome sequencing, and Sanger sequencing, Lu and colleagues [37] established *DZIP1L,* located on chromosome 3 (3q22.1-q23) (Figure 2A), and encoding a 767-amino acid protein as a new gene related to ARPKD pathogenesis. The authors identified mutations in seven patients (~0.3% of their cohort) with the ARPKD phenotype without evidence of another mutation in the PKD genes. They identified homozygous missense mutations that segregate with the disease in two families (p. Gln91His and p.Ala90Val) and homozygous protein-truncating mutations in two other unrelated consanguineous pedigrees (p.Gln155* and p.Glu354Alafs*39). The data suggest that *DZIP1L* mutations are not a common cause of the disease, but despite this fact, ARPKD NGS diagnostic multigene panels should target this gene for two reasons: mutations in *DZIP1L* may interact with other PKD or ciliopathy loci and would help to broaden the genetic complex understanding of the disease [37,53].

### Genotype-Phenotype Correlation

Establishing a possible genotype/phenotype correlation is complicated by compound heterozygotes. The most common mutation, c.107C>T (T36M), only explains about 20% of mutant alleles, but no others are described as a cluster; in fact, many variants are unique to single lineages. There are plenty of pathogenic variants in *PKHD1*, including truncating, missense, and intronic/splice mutation. Typically genotype-phenotype studies are done regarding the type of mutation more than the specific site of the mutation [54,55].

Patients with two truncating mutations show a severe phenotype with high peri- or neonatal mortality and the presence of one or two missense mutations generally exhibits a moderate phenotype. However, there are exceptions, a patient described by Ebner and colleagues with two truncating mutations in *PKHD1* survived the first 30 months of life without renal replacement therapy or, on the contrary, several cases with two missense mutations that can be as severe as truncating variants [55,56,57,58].

Furthermore, up to 20% of siblings show a marked variation in phenotype, which means that the genotype is insufficient to explain the phenotypic variability in ARPKD, where complex transcriptional profiles may play an important role [54,59]. Moreover, there is evidence in ARPKD mouse models with variants in other genes that the disease is modified; for example, co-inheritance of mutations in *Pkhd1* and *Pkd1* worsens the phenotype; this correlates in humans where a mutation in the other ADPKD gene (*PKD2*) also worsen the kidney manifestation [60,61]. It is also thought that the epigenetics and environmental factors, as well as genetic variants in other genes associated with PKD, could explain the inter-and intrafamilial variability.

## 6. ARPKD Proteins: Structure and Function

The protein product of *PKHD1* is fibrocystin/polyductin/FPC (Figure 2B) [34,48], a membrane protein with a long extracellular N-terminus, a single transmembrane domain and a short cytoplasmic C-terminus tail. The extracellular domain contains twelve TIG/IPT domains (Ig-like domains) that have been described in cell surface receptors [62]. In addition, three potential protein kinase A (PKA) phosphorylation sites were identified in the cytoplasmic tail that may be relevant for its function [63]. A *PKHD1* homologue was reported, *PKHD1L*, with an identity of 25% and similarity of 41.5%, which encodes fibrocystin-L, a receptor with inducible T lymphocyte expression, and has not been implicated in PKD [64]. The longest open reading frame (ORF) of FPC is predicted with a length of 4074 amino acids [65]. However, the *PKHD1* gene undergoes a complicated splicing pattern and can encode several additional gene products. In the same way, FPC exhibits a highly complex pattern of Notch-like proteolytic processing validated at the in vitro level [66] and in the vivo level using mouse models [67], which make the investigation of *PKHD1*/FPC particularly difficult.

FPC is a 440 kDa membrane-bound protein that is expressed mainly in the kidney (cortical and medullary ducts), the liver (intra- and extra-hepatic biliary ducts) and the pancreas (pancreatic ducts) [65,68,69]. Two alternative FPC products of ~230 and ~140 kDa were detected and, more importantly, the ~140 kDa product was found in cellular fractions of secreted FPC products [65]. At the subcellular level, FPC is expressed in the primary apical cilia [65,68,70] and the basal body area of cilia [69] in renal epithelial cells and cholangiocytes [71]. Furthermore, FPC is also expressed in the apical membrane and cytoplasm of collecting duct cells [65]. It is controversial whether ARPKD tissues lack FPC expression, some studies support this idea [48,70], but other evidence suggests otherwise [72], suggesting a temporal and spatial expression complexity of FPC splicing variants.

The structure of FPC suggests a possible function of the cell surface receptor, which interacts with extracellular ligand through the N-terminus or transduces intracellular signals to the nucleus through its C-terminus [73]. The cytoplasmic tail can translocate to the nucleus after full-length cleavage [66,74]. However, the intrinsic mechanism of the C-terminus remains unclear, as its deletion in mouse models did not result in renal or hepatic cystic phenotype, suggesting that it is not essential for cyst formation in ARPKD [67].

*DZIP1L* encodes the DAZ (Deleted in AZoospermia) interacting protein 1-like, a zinc-finger protein with several coiled-coil domains and one C2H2-type zinc finger domain near its N-terminus [37]. The zinc finger protein DZIP1L is involved in primary cilium formation [75], and Lu and colleagues suggest a possible function in the polycystins/PCs (the ADPKD proteins) trafficking [37]. The results highlighted the transition zone of cilia as a new possible vital point to study ARPKD pathogenesis [53].

## 7. Pathogenesis of ARPKD/Molecular Basis/Disease Mechanism

### 7.1. ARPKD Rodent Models: Lessons from Animal Models

To date, several animal models have been developed in which they closely resemble human ARPKD (Table 1). Early models of PKD resulted from spontaneous mutations in non-orthologous genes that mimic the recessive trait and phenotype of the disease [76]. The first mouse model reported was the congenital polycystic kidney mouse, or *cpk*, in 1985 [77]. The development and expression/penetrance of disease and the genetics in this model were extensively studied [76,78]. The *cpk* model results in a spontaneous mutation in the C57BL/6J (B6) strain, which corresponds with the *Cys1* gene [62]. Later, during the 1990s, other models appeared with spontaneous mutations in other loci, including the well-studied *pcy* mouse [79] that has a mutation in the locus for *Nphp3* [80]. Furthermore, BALB/c polycystic kidney (*bpk*) [81] and the juvenile cystic kidney model (*jck*) [82] were described and characterized, that had spontaneous mutations in *Bicc1* [83] and *Nek8* [84] respectively. This was followed by animal models designed by chemical induction, as the juvenile congenital polycystic kidney (*jcpk*), which was obtained using a chlorambucil mutagenesis program [85]. Interestingly, later studies showed that *bpk* and *jcpk* models refined the mutated loci in *Bicc1* gene [83,86]. Furthermore, by insertional mutagenesis, the Oak Ridge polycystic kidney or *orpk* mouse was uncovered from a large-scale insertional mutagenesis program [87,88].

In the 2000s, and with the discovery of *PKHD1* as the main ARPKD gene [47,48], the first ARPKD animal models appeared. The polycystic kidney rat or PCK rat was initially proposed as ADPKD model due to its slow progressive kidney and liver disease [89], but later was confirmed that the *Pkhd1* gene was disrupted in the PCK rat [48]. The first *Pkhd1*-based transgenic mouse model was *Pkhd1^ex40^* [90], and later, many others appeared, as well as a *Dzip1l*-based model (Table 1). Remarkably, the hepatic ARPKD-like phenotype was always present in all *Pkhd1*-based models, but the renal phenotype was often absent. Interestingly, pancreatic cysts were often present in these models, unlike in patients with ARPKD [6].

The key or main molecular mechanism of cystogenesis in ARPKD remains unknown. Nevertheless, animal models have allowed us to expand our understanding of the different stages of the disease, from cyst formation to cyst progression. Throughout this complex process, several altered molecular pathways such as fluid secretion, abnormal cellular proliferation (such as mTOR, RAS-RAF-ERK and AKT), cAMP pathway regulated by PKA kinase and AC6, alterations in extracellular matrix (ECM), among others have been described [91,92]. Therefore, the understanding of the pathophysiology of ARPKD has improved in recent decades thanks to the existence of a good variety of animal models. However, the key intrinsic molecular mechanism of cystogenesis in ARPKD remains unknown, leading to increased interest in understanding the mechanism of the disease and developing new therapeutic strategies. Next, we review the main molecular pathways characterized in ARPKD.

**Table 1:** List of current animal models of ARPKD, or that mimics it phenotype. Animal models were ordered from least to most recent, according to published data.

Model Name	Specie	Gene	Allele Type (Mutation Type)	Liver Phenotype	Kidney Phenotype	Other Phenotypes	Ref.
Animal models that mimics ARPKD							
*cpk* (*Cys1^cpk^*) *	Mouse	*Cys1*	Spontaneous	∙Liver cysts ∙Dilated bile duct ∙Hepatic fibrosis	∙Kidney cysts ∙Enlarged kidney	∙Pancreas cysts	[77,93,94,95,96]
*pcy* (*DBA/2-pcy/pcy*) (*Nphp3^pcy^*) *	Mouse	*Nphp3*	Spontaneous	None	∙Kidney cysts ∙Enlarged kidney ∙Renal fibrosis	∙Intracranial aneurysm	[79,80,97]
*bpk* (*Bicc1^jcpk-bpk^*) *	Mouse	*Bicc1*	Spontaneous	∙Enlarged bile duct	∙Kidney cysts ∙Enlarged kidney	∙Premature death ∙Postnatal lethality	[81]
jck (*Nek8^jck^*) *	Mouse	*Nek8*	Spontaneous	∙None	∙Kidney cysts ∙Enlarged kidney	∙Premature death	[82]
orpk (*Ift88^Tg737Rpw^*) *	Mouse	*Ift88*	Transgenic (insertion)	∙Abnormal bile duct morphology ∙Liver fibrosis	∙Kidney cysts ∙Enlarged kidney	∙Pancreas cysts ∙Polydactyly	[87,88]
*jcpk*	Mouse	*Bicc1*	Chemical induction	∙Dilated bile duct	∙Kidney cysts ∙Dilated renal tubules	∙Dilated pancreatic ducts	[85]
ARPKD models							
PCK	Rat	*Pkhd1*	Spontaneous (splicing mutation)	∙Liver cysts ∙Dilated bile duct ∙Hepatic fibrosis	∙Kidney cysts ∙Enlarged kidney ∙Renal fibrosis	∙Pancreas cysts	[89,98]
Pkhd1ex40	Mouse	*Pkhd1*	Targeted (KO by insertion)	∙Liver cysts ∙Hepatic fibrosis	∙None	∙Portal hypertension	[90]
*Pkhd1^del2/del2^* (*Pkhd1^tm1Cjwa^*) *	Mouse	*Pkhd1*	Targeted (KO by deletion)	∙Liver cysts ∙Dilated bile duct ∙Hepatic fibrosis	∙Kidney cysts ∙Dilated renal tubules	∙Pancreas cysts ∙Pancreatic duct abnormalities	[99]
*Pkhd1^del3−4^* (*Pkhd1^tm1^.^1Ggg^*) *	Mouse	*Pkhd1*	Targeted (KO by deletion)	∙Liver cysts ∙Hepatic fibrosis	∙Kidney cysts ∙Renal fibrosis	∙Pancreas cysts ∙Choledochal cyst ∙Ascending cholangitis	[60]
*Pkhd1^del4/del4^* (*Pkhd1^tm1Som^*)*	Mouse	*Pkhd1*	Targeted (KO by deletion)	∙Liver cysts ∙Biliary cysts ∙Hepatic fibrosis	∙None	∙Pancreas cysts ∙Pancreas fibrosis ∙Enlarged spleen	[100]
*Pkhd1^e15GFP^^∆^^16^* (*Pkhd1^tm1Gwu^*) *	Mouse	*Pkhd1*	Targeted (KO by deletion)	∙Liver cysts ∙Hepatic fibrosis	∙Kidney cysts ∙Dilated renal tubules ∙Renal fibrosis	∙Pancreas cysts ∙Dilated pancreatic duct ∙Gastrointestinal ulcer	[101]
*Pkhd1^lacZ^* (*Pkhd1^tm1Sswi^*) *	Mouse	*Pkhd1*	Targeted (KO by deletion)	∙Dilated bile duct ∙Hepatic fibrosis	∙Kidney cysts ∙Dilated renal tubules ∙Renal fibrosis	∙Pancreas cysts	[102]
*Pkhd1^LSL(-)^* (*Pkhd1^tm2Cjwa^*) *	Mouse	*Pkhd1*	Targeted (KO by insertion)	∙Liver cysts ∙Hepatic fibrosis	∙Kidney cysts ∙Dilated renal tubules	∙Unknown	[103]
*Pkhd1^Δ67^*	Mouse	*Pkhd1*	Targeted (KO by deletion)	∙None	∙None	∙None	[67]
*Dzip1l^wpy/wpy^* (*Dzip1l^warpy^*) *	Mouse	*Dzip1l*	Targeted (KO by single point mutation)	∙Abnormal bile duct morphology	∙Kidney cysts ∙Dilated renal tubules	∙Polydactyly ∙Abnormal eye morphology ∙Cleft upper lip ∙Cleft palate	[37]
Pkhd1^C642*^ (*Pkhd1^em1Mrug^*) *	Mouse	*Pkhd1*	Targeted (KO by deletion)	∙Liver cysts ∙Biliary cysts ∙Hepatic fibrosis	∙Dilated renal tubules ∙Proximal tubule ectasia	∙Unknown	[67]

* Model name according to MGI (Mouse Genome Informatics) [104]. Ref. = reference. KO = knockout.

### 7.2. Abnormalities of EGFR-Axis Expression and Fluid Secretion

**Figure 3:**
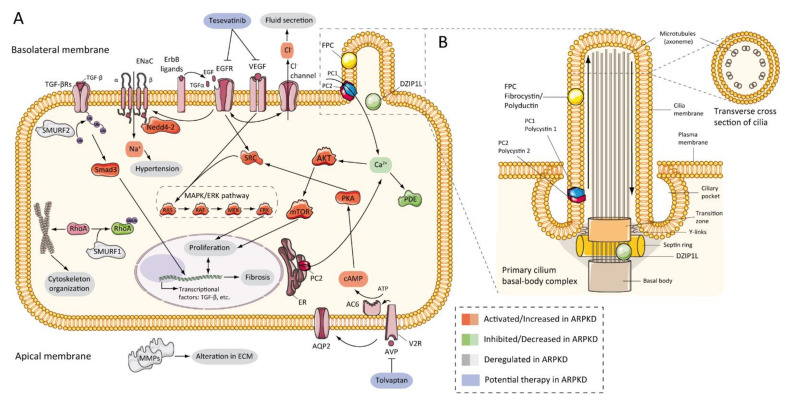
ARPKD molecular pathogenesis: (**A**) diagram representing the proposed up-, down-, or deregulated pathways in ARPKD renal epithelial cell and proposed potential therapies; (**B**) cartoon representing the localization of ARPKD protein in the cilium of a renal epithelial cell. FPC is located in the primary apical cilia and the basal body area of the cilia, whereas DZIP1L is located in the transition zone of the basal body of the cilium. (FPC—fibrocystin/polyductin; DZIP1L—DAZ interacting zinc finger protein 1 like; PC1—polycystin 1; PC2—polycystin 2; TGF—transforming growth factor; ENaC—epithelial sodium channels; Na^+^—sodium cation; EGFR—epidermal growth factor receptor; VEGF—vascular endothelial growth factor; Cl^−^—chlorine anion; SMURF—SMAD specific E3 ubiquitin-protein ligase; Nedd4-2—E3 ubiquitin-protein ligase Nedd4-2; SRC—proto-oncogene tyrosine-protein kinase Src; AKT—RAC-alpha serine/threonine-protein kinase; Ca^+^—calcium cation; PDE—phosphodiesterase; mTOR—mammalian target of rapamycin; MAPK—mitogen-activated protein kinases; RAF—rapidly accelerated fibrosarcoma; MEK—mitogen-activated protein kinase kinase; ERK—extracellular-signal-regulated kinase; Rhoa—Ras homolog family member A; ER—endoplasmic reticulum; PKA—protein kinase A; cAMP—cyclic adenosine monophosphate (cyclic AMP); ATP—adenosine triphosphate; AC6—adenylate cyclase type 6; AQP2—aquaporin 2; V2R—vasopressin receptor 2; AVP—arginine vasopressin; MMPs—matrix metalloproteinases).

The first evidence that the epidermal growth factor receptor (EGFR) axis was altered in PKD was in 1992, by demonstrating that cells from primary cultures of PKD patients increased cyst expansion [105]. Subsequently, in primary cells isolated from ADPKD patients, epidermal growth factor (EGF) stimulated cyst formation [106]. In ARPKD, the first data were obtained from *cpk* mouse model renal extracts, which showed upregulation of EGF expression [107]. Progressively, other evidence has shown a significant role for EGFR in vitro [108] and murine models [88,109,110], and patients with ARPKD [111,112], where EGFR upregulation was located on the surface of the cystic epithelium. In the same way, abnormal expression of EGF [113,114] and transforming growth factor-alpha (TGFα) [115] have been demonstrated in ARPKD, and several members of EGFR family of receptors (EGFR1, ErbB2, and ErbB4) were found overexpressed in ARPKD rodent models [72,116] (Figure 3A). This overexpression includes increased mRNA, protein, and receptor activity or phosphorylation [92]. Furthermore, evidence from animal models suggests similar abnormalities in hepatic cystogenesis of the EGFR axis [117].

EGFR signaling has relevance in cystogenesis, a correlation between upregulation of EGFR tyrosine kinase activity and cystic growth. A modified *orpk* model with a point mutation that decreased EGF tyrosine kinase activity (wa2 mouse) [118], showed a significant decrease in cyst formation and improvement of renal function [110]. Furthermore, pharmacological inhibition with EGFR-specific tyrosine kinase inhibitors caused a decreased EGFR activity leading to a significant reduction of cyst progression [119,120,121]. Controversially, the EGFR tyrosine kinase inhibitor in the PCK rat did not slow the progression of renal cysts [122].

Epithelial secretion is a key physiopathology component of cyst formation. Reduced sodium uptake in ARPKD net fluid secretion has been proposed as being related to decreased EGF in the alpha subunit of the epithelial Na channel [123,124]. Nevertheless, conflicting data have been published on this topic [116]. In cells derived from cysts from patients with ARPKD, it was suggested that sodium absorption was mediated by the epithelial sodium channel (ENaC) [125]. Furthermore, this mechanism has been proposed as a regulator of hypertension in ARPKD [125,126,127].

On the other hand, in ADPKD, the data show that Cl^−^ secretion occurs through the cystic fibrosis transmembrane conductance regulator (CFTR) [128,129]. However, in ARPKD this mechanism has no apparent relevance. In the *bpk* murine model, the absence of CFTR did not slow the progression of the renal and hepatic cyst [130]. These data suggest that the mechanisms of Cl^−^ and fluid secretion are different in ADPKD and ARPKD.

### 7.3. cAMP and Proliferation

Several studies have shown that adenylyl cyclase adenosine 3′,5′-cyclic monophosphate (cAMP) pathway stimulates cell proliferation in the renal epithelium of ARPKD and ADPKD. Production of cAMP is aberrant in the cyst epithelium, resulting in a large amount of this nucleotide in the cyst fluid [122,131,132,133]. cAMP activates the B-Raf, MEK, and ERK pathways in the cyst epithelium of the kidneys with ADPKD [134,135,136], and ADPKD and ARPKD cells in culture [133]. In the same way, these results were complemented with data showing upregulation at the protein level of MAPK and AKT/mTOR pathways in several rodent models of ARPKD [137,138,139,140,141]. These facts correlated with the reduction of intracellular Ca^2+^ and the phosphorylation of the SCR protein [142,143,144]. In particular, blocked intracellular Ca^2+^ elevated AKT and proliferative activity in ARPKD cells in culture [143]. This study opened the opportunity to use the level of intracellular calcium restoration as a therapeutic approach in PKD.

Dysregulation of calcium in PKD causes upregulation of the vasopressin V2 receptor [136,138], which activates the cAMP/PKA cascade [145,146]. In a preclinical trial, the V2 receptor antagonist has demonstrated its efficacy in ARPKD rat model, reducing the renal cAMP levels and improving cystic renal disease [137] (Figure 3A). This fact has been further studied, reviewed, and understood in ADPKD, at preclinical [147,148] and clinical levels [149], to the point that Tolvaptan (an vasopressin V2 receptor inhibitor) was approved for use in human patients [150] and an ongoing trial for children with ADPKD (Tolvaptan (NCT02964273)) [151]. ARPKD-related clinical trials will be reviewed later.

### 7.4. Other Pathways Involved in ARPKD Physiopathology

Other pathogenic features have been identified in ARPKD, as well as alterations in extracellular matrix (ECM) and metalloproteinase expression (MMPs) [152,153], upregulation of vascular endothelial growth factor (VEGF) and hypoxia-inducible factor-1 alpha (HIF-1α) in *Pkhd1* deficient cells [139], upregulation of peroxisome-proliferator-activated receptor-γ (PPAR-γ) in animal models [154,155], or metabolic alterations [156]. In a large and interesting study, Kaimori and colleagues published information about novel functional relationships between FPC and members of the C2-WWW-HECT domain E3 family of ubiquitin ligases. The authors localized FPC in vesicles where Ndfip2 was also present, a ubiquitin ligase interacting protein implicated in trafficking and regulating the Nedd4-2 ubiquitin ligase family and SMURF1 and SMURF2. These data may explain different universal phenotypes in ARPKD and renal and hepatic fibrosis through TGF-β signaling pathways, hypertension through to ENaC mediated sodium reabsorption, and cystogenesis through to RhoA ubiquitination and cytoskeleton organization [127] (Figure 3A). In other studies, tubular morphogenesis in PKD was associated with an abnormality planar cell polarity (PCP) [157]. However, later studies have shown contrary results [158].

### 7.5. Role of Cilia

Figure 3 shows the ARPKD proteins (zinc finger protein DZIP1L and FPC) are located in the cilia. The cilia are long and microtubular structures emanating from the surface of mammalian cells. The axoneme of primary cilia contains nine peripheral bundles of microtubules (9 + 0 pattern). Pathologies related to a loss of proper cilia function are called ciliopathies, including ARPKD [159]. PC2, also called TRPP2, is a member of the transient receptor channel (TRP) family and is a calcium-permeable non-selective channel [160]. PC2 and PC1 form a receptor-channel complex, that is involved in the calcium pathway and cilia response [161,162,163] (Figure 3B). FPC has been shown to interact with PC2 in primary cilia and regulates PC2 channel activity [101,164,165]. In addition, it has been reported that the C-terminus of FPC physically interacts with the N-terminus of PC2 in vivo and in vitro, and that *Pkhd1*-deficient cells exhibit dysregulation of PC2 channel activity [101]. However, Wang and colleagues found no differences in PC2 levels in cells with reduced FPC levels [165]. Other data using a novel *Pkhd1* mouse model have shown that deletion of the last exon of *Pkhd1*, the PC2 binding site, and the nuclear localization signal, had no apparent pathologic effects in mice [67]. In addition, the researchers were unable to co-precipitate FPC-PC2 in kidney samples from the transgenic mouse model. These results suggest that the PC2 binding domain of FPC is not essential for the fibrocystin function [67,166].

We have described a genetic interaction between *Pkd1* and *Pkhd1*, linking ADPKD and ARPKD [60]. Consequently, additional data has reported this genetic interaction between *Pkd1* and *Pkhd1* in other rodent models, describing mild cystic disease phenotypes of *Pkd1* and/or *Pkhd1* enhanced their severity in combination [38,167]. These studies and others have expanded the relationship between FPC and PC1. Using in-vitro models, the loss of FPC did not affect the biogenesis and location of PC1, suggesting that the genetic interaction between *Pkd1* and *Pkhd1* is indirect [30,167].

The results of these studies appear consistent with the idea that the PKD proteins form a functional complex in cilia with common downstream signaling pathways [168]. Interestingly, cilia loss suppresses renal cyst growth in murine models of ADPKD and autosomal dominant polycystic liver disease (ADPLD) murine models [169]. However, in a recent study, Gallagher and Somlo reported that this loss of cilia does not slow the progression of liver disease in ARPKD [170]. These data suggest that ADPKD and ARPKD, at least in the hepatic cystic phenotype, do not share a common cilia-related pathway.

On the other hand, the DAZ interacting protein 1-like protein (DZIP1L), based on several cell culture studies, zebrafish, and mice, localizes in centrioles and in the ciliary transition zone the primary cilia [37]. Lu and colleagues have demonstrated the interaction between DZIP1L and septin 2 (SEPT2), a protein involved in the maintenance of the periciliary diffusion barrier at the transition zone [171] and the co-localization with tectonic 1 (TCTN1), a ciliary transition zone protein. In *DZIP1L* mutant cells, the transport of polycystin-1 and -2 from the basal body to the axoneme of the cilia was altered; both were retained in the basal body when their normal distribution was in the ciliary axoneme. However, *DZIP1L* deficiency did not alter the localization or expression of FPC [37]. These findings suggest a role of *DZIP1L* in the trafficking of polycystins and new evidence that links ARPKD with ADPKD.

## 8. Clinical Trials

As we have noted, the central or key mechanism of cystogenesis in PKD remains unclear. There is evidence of several pathways involved in the pathogenesis of PKD from cellular and animal studies. These facts have allowed several drugs to reach the clinical phase (Table 2).

Based on the results of phase 3 and 4 clinical trials [149,172,173], two clinical trials are currently underway using the pharmacological intervention of Tolvaptan. The primary objective of these trials is to evaluate the safety of Tolvaptan in infants (8 days or less) and children (less than 18 years) (NCT04782258) and in pediatric patients (from 28 days to 12 weeks of age) (NCT04786574). On the other hand, PKD exhibits an abnormal c-Src activity, links the cAMP and EGFR molecular ways [174], and its inhibition ameliorates renal cystogenesis [144]. These data led Sweeney et al. to study the effect of a multi-kinase inhibitor of EGFR axis, c-Src and VEGFR, called tesevatinib (TSV), as a possible therapy in preclinical studies for ARPKD obtaining favorable results [175]. The positive and promising results led to the approval of phase I and II of TSV clinical trials for ARPKD (NCT03096080). Finally, two observational trials are being carried out to expand the knowledge of the disease (genotype-phenotype correlations, clinical aspects) and to create more precise mutational and clinical databases (NCT01401998 and NCT00068224).

## 9. Conclusions

The ARPKD field has experienced significant progress in the areas of genetics, diagnostics, and molecular biology. First, the identification of the *PKHD1* gene, for several years considered the only ARPKD gene, and more recently the *DZIP1L* gene, and its implementation in genetic diagnosis based on next-generation sequencing (NGS). Second, advances in the understanding of the function and localization of the fibrocystin/polyductin protein. Finally, different studies, especially in animal models, begin to elucidate the pathways involved in the pathogenesis of the disease identifying possible therapeutic approaches.

However, many unanswered questions should be answered in the future. The new finding of *DZIP1L* as a second genetic locus for ARPKD leaves open the possibility of the appearance of new genes that cause ARPKD in this regard it is necessary to apply genetically unresolved whole exome sequencing (WES) families (GUR) with phenotype ARPKD. The exact function of the FPC remains unknown, as well as a correct characterization of all its isoforms. Importantly, the key factor(s) driving cyst formation in PKD are not clear. For these reasons, the pathogenicity of ARPKD is poorly understood and, even today, there is no approved therapy for existing replacement therapy. To explain the genetic and cellular basis of ARPKD, research on the subject emerges as the only way out of this situation.

## Figures and Tables

**Table 2:** Currently active or complete clinical trials for ARPKD.

Identifier	Intervention	Study Design and Characteristics	Study Description	Sponsor
NCT04782258	Tolvaptan	∙Study type: interventional ∙Primary purpose: treatment ∙Period: April 2021–June 2025 (estimated) ∙Patients: 20 (estimated, not yet recruitment) ∙Allocation: non-randomized ∙Intervention model: parallel assignment ∙Masking: none (open label)	∙The primary objective of this phase 3 trial is to evaluate the safety of Tolvaptan (OPC-41061) in infants and children, 8 days to less than 18 years old of age, with ARPKD. ∙Participants in this study will be assigned to Tolvaptan for 18 months and closely monitored over the course of the study.	Otsuka Pharmaceutical Development & Commercialization, Inc. Princeton, New Jersey, USA.
NCT04786574	Tolvaptan	∙Study type: interventional ∙Primary purpose: treatment ∙Period: April 2021–July 2025 (estimated) ∙Patients: 20 (estimated, not yet recruitment) ∙Allocation: N/A ∙Intervention model: single group assignment ∙Masking: none (open label)	∙In this Phase 3 trial, the primary objective is to evaluate safety, tolerability, and efficacy of Tolvaptan (OPC-41061) in pediatric subjects, 28 days to less than 12 weeks of age, with ARPKD. ∙Participants in this trial will be assigned to Tolvaptan for 24 months and closely monitored over the course of the study.	Otsuka Pharmaceutical Development & Commercialization, Inc. Princeton, New Jersey, USA.
NCT03096080	Tesevatinib	∙Study type: interventional ∙Primary purpose: treatment ∙Period: March 2017–October 2019 (completed) ∙Patients: 10 ∙Allocation: Non-randomized ∙Intervention model: sequential assignment ∙Masking: none (open label)	∙This trial in Phase 1 evaluates safety and tolerability of a single ascending dose of a Tesevatinib (KD019, XL647) liquid formulation administered to pediatric subjects (child with age 5–12 years) with ARPKD. ∙To determine safety of the Tesevatinib liquid formulation in pediatric subjects with ARPKD, all participants receive active study drug on Day 1 of the study enrollment.	Kadmon Corporation, LLC Philadelphia, Pennsylvania, USA. Milwaukee, Wisconsin, USA.
NCT01401998	Observational	∙Study type: observational ∙Period: July 2011–December 2022 (recruitment) ∙Patients: 200 (estimated) ∙Observational model: cohort ∙Time perspective: retrospective assignment	∙This study captures clinical and genetic information of ARPKD patients to expand the knowledge of disease. ∙The primary goal of this trial is create a clinical and mutational databases including clinical information and identifying genetic mutations from all patients enrolled in the study. ∙Mutational database will be useful to facilitate genetic research as genotype-phenotype correlations, new disease gene studies, or modifier gene studies. ∙Create a tissue resource with human tissue from both affected and controls individuals.	Lisa M. Guay-Woodford (Collaborator: National Institute of Diabetes and Digestive and Kidney Diseases (NIDDK)). Washington, District of Columbia, USA.
NCT00068224	Observational	∙Study type: observational ∙Period: September 2003–February 2021 (completed) ∙Patients: 374 ∙Observational model: cohort ∙Time perspective: prospective	∙This study evaluated patients with ciliopathies, including ARPKD. ∙ The goal of the study is to better understand the medical complications of these disorders and identify characteristics that can help in the design of new treatments.	National Human Genome Research Institute (NHGRI). Bethesda, Maryland, USA.

Status according to https://clinicaltrials.gov/, accessed on 6 April 2021.
